# Genetic visualization of the secondary olfactory pathway in Tbx21 transgenic mice

**DOI:** 10.1186/2042-1001-1-5

**Published:** 2011-02-01

**Authors:** Sachiko Mitsui, Kei M Igarashi, Kensaku Mori, Yoshihiro Yoshihara

**Affiliations:** 1 Laboratory for Neurobiology of Synapse, RIKEN Brain Science Institute, 2-1 Hirosawa, Wako-shi, Saitama 351-0198, Japan; 2 Department of Physiology, Graduate School of Medicine, The University of Tokyo, 7-3-1 Hongo, Bunkyo-ku, Tokyo 113-0033, Japan; 3 Kavli Institute for Systems Neuroscience and Centre for the Biology of Memory, Norwegian University of Science and Technology, Trondheim, Norway

## Abstract

**Background:**

Mitral and tufted cells are the projection neurons in the olfactory bulb, conveying odour information to various regions of the olfactory cortex. In spite of their functional importance, there are few molecular and genetic tools that can be used for selective labelling or manipulation of mitral and tufted cells. Tbx21 was first identified as a T-box family transcription factor regulating the differentiation and function of T lymphocytes. In the brain, Tbx21 is specifically expressed in mitral and tufted cells of the olfactory bulb.

**Results:**

In this study, we performed a promoter/enhancer analysis of mouse *Tbx21 *gene by comparing nucleotide sequence similarity of *Tbx21 *genes among several mammalian species and generating transgenic mouse lines with various lengths of 5' upstream region fused to a fluorescent reporter gapVenus. We identified the *cis*-regulatory enhancer element (~300 nucleotides) at ~ 3.0 kb upstream of the transcription start site of *Tbx21 *gene, which is both necessary and sufficient for transgene expression in mitral and tufted cells. In contrast, the 2.6-kb 5'-flanking region of mouse Tbx21 gene induced transgene expression with variable patterns in restricted populations of neurons predominantly located along the olfactory pathway. Furthermore, we generated transgenic mice expressing the genetically-encoded fluorescent exocytosis indicator, synaptopHluorin, in mitral and tufted cells for visualization of presynaptic neural activities in the piriform cortex.

**Conclusions:**

The transcriptional enhancer of *Tbx21 *gene provides a powerful tool for genetic manipulations of mitral and tufted cells in studying the development and function of the secondary olfactory pathways from the bulb to the cortex.

## Background

Odour molecules emitted from objects enter into the nostrils, reach the olfactory epithelium, and bind odourant receptors (ORs) expressed on the cilia of olfactory sensory neurons (OSNs). The odour information is then converted into electrical signals, transmitted to glomeruli in the olfactory bulb (OB) via precisely wired neural circuitry and represented as topographic 'odour maps' on the glomerular array of the OB [1]. After the OR multigene family was discovered [2], the basic principles of olfactory axon wiring to the glomeruli were elucidated using molecular biological, genetic engineering, electrophysiological and neural activity imaging studies [3-9]. In particular, the availability of various molecular markers and transcriptional enhancers has facilitated the distinguishing, labelling and manipulating of distinct types of OSNs. This has greatly promoted the recent progress in the understanding of the primary olfactory system [10-14].

In contrast, little is known of the functional architecture of axonal projections that extend from the OB to the olfactory cortex in mammals. For example, it is not known how odour information is transmitted from the OB to various regions of the olfactory cortex, including the piriform cortex, the cortical amygdaloid nuclei and the lateral entorhinal cortex, or how the topographic OR map on the OB is decoded in the olfactory cortex for the translating of odour inputs into olfactory perception, emotion, memory and behavioural responses. Mitral and tufted cells are the excitatory projection neurons in the OB that relay the odour information from glomeruli to various regions of the olfactory cortex. Despite their functional significance, only a few molecular genetic tools are available for the selective labelling or manipulation of mitral and tufted cells [15-17].

In this study, we focused on the mouse *Tbx21 *(*T-bet*) gene, which is specifically expressed in mitral and tufted cells in the brain [18]. We analysed transgenic mice that harboured transgenes with various lengths of Tbx21 5'-upstream regions fused to a fluorescent reporter. As a result, we identified a *cis*-regulatory enhancer element that is necessary and sufficient for transgene expression in mitral and tufted cells. Furthermore, the enhancer was utilized to visualize presynaptic neural activities in the olfactory cortex by generating transgenic mice that expressed the genetically-encoded fluorescent exocytosis indicator, synaptopHluorin (spH), in mitral and tufted cells.

## Results

### Mitral and tufted cell-specific expression of Tbx21 protein in the mouse brain

**Figure 1:**
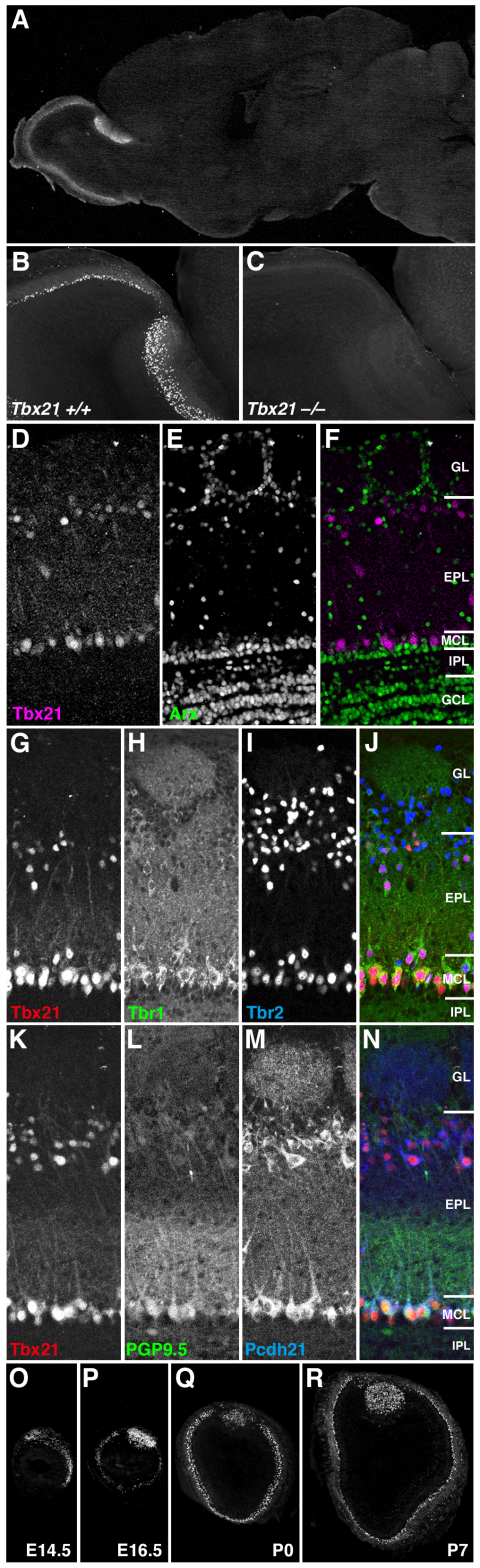
**Mitral and tufted cell-specific expression of Tbx21 protein**. (A) A parasagittal section of adult mouse brain labelled with anti-Tbx21 antibody. Strong fluorescent signals are specifically observed in the olfactory bulb (OB). (B) A higher magnification of the OB. (C) No immunoreactivity in the OB section from Tbx21-deficient mouse. (D-F) Double immunofluorescence labelling of OB section with anti-Tbx21 (D, F (magenta)) and anti-Arx (E and F (green)) antibodies. Tbx21 protein is expressed in the mitral and tufted cells but not in Arx-positive interneurons. (G-J) Triple immunofluorescence labelling of OB section with anti-Tbx21 (G and J (red)), anti-Tbr1 (H and J (green)) and anti-Tbr2 (I and J (blue)) antibodies. The three T-box family transcription factors are expressed in mostly overlapping, but some differential, subpopulations of mitral and tufted cells. (K-N) Triple immunofluorescence labelling of the OB section with anti-Tbx21 (K and N (red)), anti-PGP9.5 (L and N (green)) and anti-protocadherin 21 (Pcdh21) (M and N (blue)) antibodies. All three molecules are expressed in the mitral and tufted cells but localized to different subcellular compartments (Tbx21 in nuclei; PGP9.5 in cell bodies and proximal dendrites; Pcdh21 in cell bodies and glomeruli). (O-R) Developmental expression of Tbx21 protein in OB coronal sections from E14.5 (O), E16.5 (P), P0 (Q) and P7 (R) mice. EPL, external plexiform layer; GCL, granule cell layer; GL, glomerular layer; IPL, internal plexiform layer; MCL, mitral cell layer.

It was previously reported that *Tbx21 *messenger RNA (mRNA) is expressed specifically in the mitral and tufted cells in the developing mouse brain [18]. We generated polyclonal antibodies in a guinea pig against a carboxyl-terminal 20-amino acid sequence of mouse Tbx21 [19] and analysed the distribution of Tbx21 protein in the developing and adult brains by immunohistochemistry. Tbx21 protein was specifically localized to the mitral and tufted cells in the OB, while no expression was observed in any other cell types in the brain (Figure 1A and 1B). The expression level of Tbx21 protein in the mitral and tufted cells was higher in the accessory OB (AOB) than that in the main OB (MOB; Figure 1B). The antibody gave no signal on the OB section prepared from *Tbx21 *knockout mouse (Figure 1C), confirming its specific reactivity to Tbx21 protein. A double immunofluorescence labelling clearly showed the presence of Tbx21 protein in the mitral and tufted cells but not in the Arx-positive GABAergic interneurons (granule and periglomerular cells; Figure 1D-F). A triple immunofluorescence labelling revealed that Tbx21 was co-localized with Tbr1, Tbr2, PGP9.5 and protocadherin-21 (Pcdh21), the four markers for mitral and tufted cells (Figure 1G-N). Quantification of the numbers of Tbx21-, Tbr1- and Pcdh21-positive cells indicated that Tbx21 is expressed in a vast majority, if not all, of the mitral cells (94.6% ± 0.6% of the Tbr1-positive mitral cells) and a large population of tufted cells (72.4% ± 3.4% of the Pcdh21-positive tufted cells) in the adult OB (*n *= 5; Additional File 1). During development, the expression of Tbx21 protein was first detectable at embryonic day 14.5, gradually increased to reach the maximal level in neonates (Figure 1O-R), and slightly decreased into adulthood (Figure 1A and 1B), consistent with the previous study showing ontogenic expression of *Tbx21 *mRNA [18].

### Faithful expression of transgene in mitral and tufted cells in Tbx5.0gV transgenic mice

**Figure 2:**
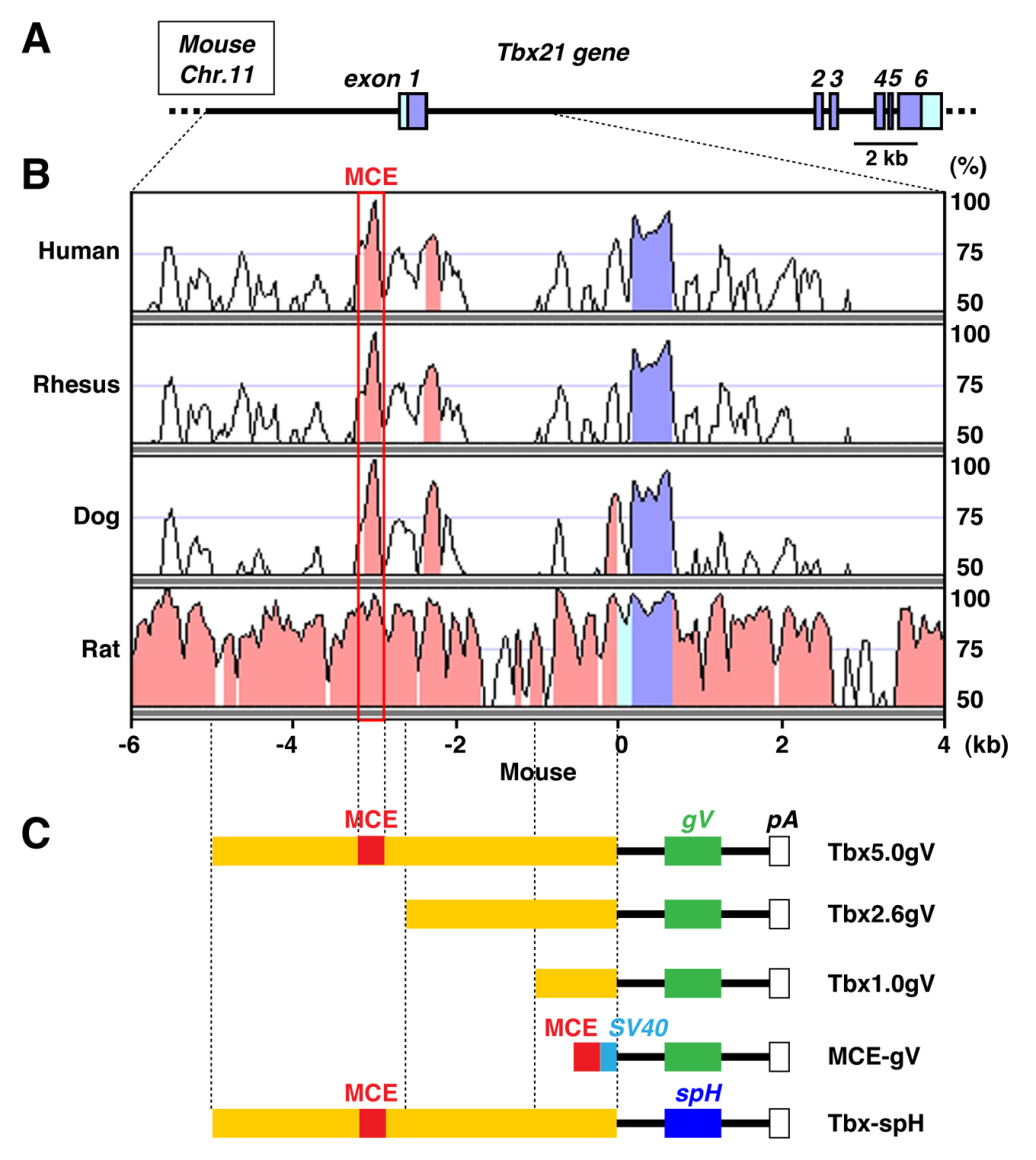
**Organization of mouse Tbx21 gene and transgene constructs**. (A) Genomic organization of Tbx21 gene on mouse chromosome 11. Exons are indicated by boxes with numbers: purple boxes indicate the open reading frame; and light blue boxes represent 5'- and 3'-non-translated regions. (B) Nucleotide sequence identity plots of human, rhesus, dog and rat Tbx21 genes compared with mouse ortholog by using the VISTA program [41]. Highly conserved regions (> 80% identity) in 5'-flanking sequences and introns are shown in pink. Mitral and tufted cell-specific enhancer (MCE; 307 bp) is present at ~3 kb upstream from the transcription start site of Tbx21 gene. (C) Transgene constructs. 5'-flanking regions of Tbx21 gene with different lengths (5.0, 2.6 and 1.0 kb: yellow) were fused to a membrane-targeted Venus complementary DNA (cDNA) (gV: green) to generate Tbx5.0gV, Tbx2.6gV and Tbx1.0gV transgenes. Highly conserved 307-bp sequence (MCE: red) and SV40 minimal promoter (blue) were fused with gapVenus cDNA to generate MCE-gV transgene. 5.0-kb 5'-flanking region of Tbx21 gene was fused with synaptopHluorin cDNA (spH: dark blue) in order to generate Tbx-spH transgene.

The *Tbx21 *gene locates on the long arm of mouse chromosome 11, a region syntenic to the human chromosome 17, and consists of six exons and five introns (Figure 2A). A homology plot revealed highly conserved nucleotide sequences in the 5'-flanking region of mouse, human, rhesus, dog and rat *Tbx21 *genes (Figure 2B), implying the presence of potential *cis*-regulatory enhancer elements. In order to identify the mitral and tufted cell-specific transcriptional enhancer, we first constructed three transgenes that carry different lengths of the 5'-flanking sequence of mouse *Tbx21 *gene (5.0, 2.6 and 1.0 kb) fused to the membrane-tethered fluorescent reporter gapVenus complementary DNA (cDNA; Figure 2C). Several founders and lines of transgenic mice were generated with each construct and analysed for gapVenus expression in the brain. The results are summarized in Additional File 2.

**Figure 3:**
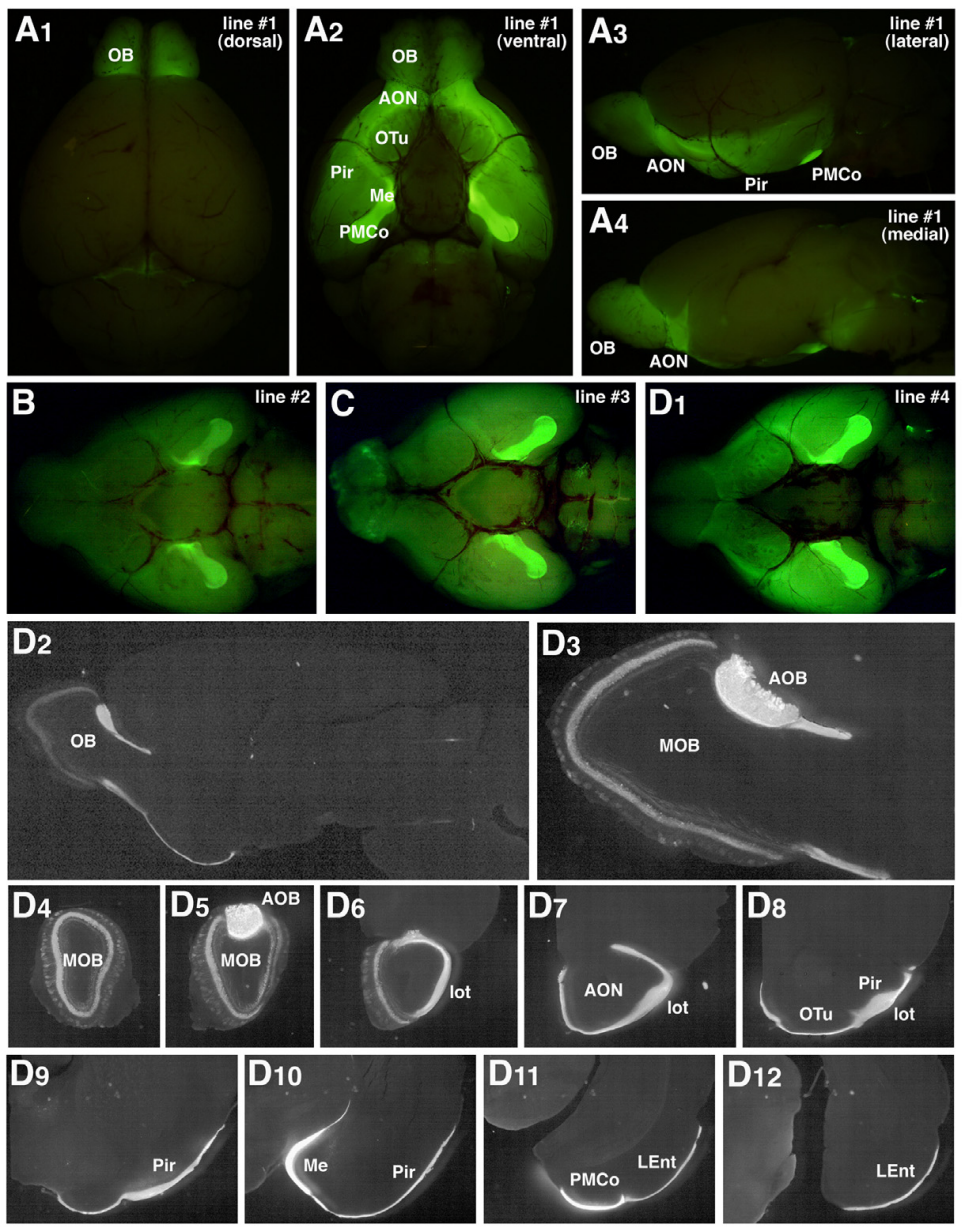
**Mitral and tufted cell-specific transgene expression in Tbx5.0gV transgenic mice**. (A_1_-A_4_) gapVenus fluorescence in whole-mount brain of Tbx5.0gV transgenic mouse line #1 viewed from dorsal (A_1_), ventral (A_2_), lateral (A_3_) and medial (A_4_) sides. Strong gapVenus fluorescence is observed in the mitral and tufted cell axons projecting from the olfactory bulb (OB) to various regions of the olfactory cortex. (B, C, D_1_) Ventral views of whole-mount brains of Tbx5.0gV line #2 (B), #3 (C), and #4 (D_1_). In all the Tbx5.0gV lines, the accessory OB (AOB) mitral/tufted cells display a higher level of gapVenus expression than the main OB (MOB) mitral and tufted cells, clearly delineating the secondary vomeronasal pathway from the AOB to the amygdaloid nuclei. (D_2_-D_12_) Parasagittal (D_2_, D_3_) and coronal (D_4_-D_12_) brain sections of Tbx5.0gV mouse line #4 immunostained with anti-green fluorescent protein antibody. (D_3_) A higher magnification of OB. AON, anterior olfactory nucleus; LEnt, lateral entorhinal cortex; lot, lateral olfactory tract; Me, medial amygdaloid nucleus; OTu, olfactory tubercle; Pir, piriform cortex; PMCo, posteromedial cortical amygdaloid nucleus.

With the longest construct harbouring 5.0-kb 5'-flanking region of the *Tbx21 *gene (Tbx5.0gV), gapVenus was faithfully expressed in the mitral and tufted cells of transgenic founders and lines at a highly efficient rate (15/17: 88%), although there are some variations in the expression patterns and/or levels which are possibly due to the positional effects of transgene integration sites at distinct chromosomal loci (Figure 3 and Additional File 1). Consistent with the difference in endogenous Tbx21 expression levels between the AOB and MOB, the transgene was expressed more robustly and frequently in the mitral and tufted cells of the AOB than those of the MOB in most transgenic lines. In ventrally viewed images of whole-mount brains, the embossed gapVenus fluorescence was observed along the secondary accessory olfactory pathway reaching the medial and posteromedial cortical amygdaloid nuclei (Figure 3 A_2_, B, C and D_1_). In order to examine the gapVenus expression in more detail, the brain sections of Tbx5.0gV mouse (line #4) were immunohistochemically stained with anti-green fluorescent protein (GFP) antibody. The gapVenus expression clearly delineated the trajectory of mitral and tufted cell axons projecting from the OB to various regions of the olfactory cortex, including the anterior olfactory nucleus, the olfactory tubercle, the piriform cortex, the medial and posteromedial cortical amygdaloid nuclei and the lateral entorhinal cortex (Figure 3D_2_-D_12_). The ontogenic expression of gapVenus transgene in the mitral and tufted cells was paralleled with that of the endogenous Tbx21, further confirming the faithfulness of this enhancer/promoter activity (Additional File 3). These results indicate that the 5.0-kb 5'-flanking region of the *Tbx21 *gene can drive transgene expression efficiently and reproducibly in the mitral and tufted cells.

### Variable patterns of transgene expression in Tbx2.6gV transgenic mice

**Figure 4:**
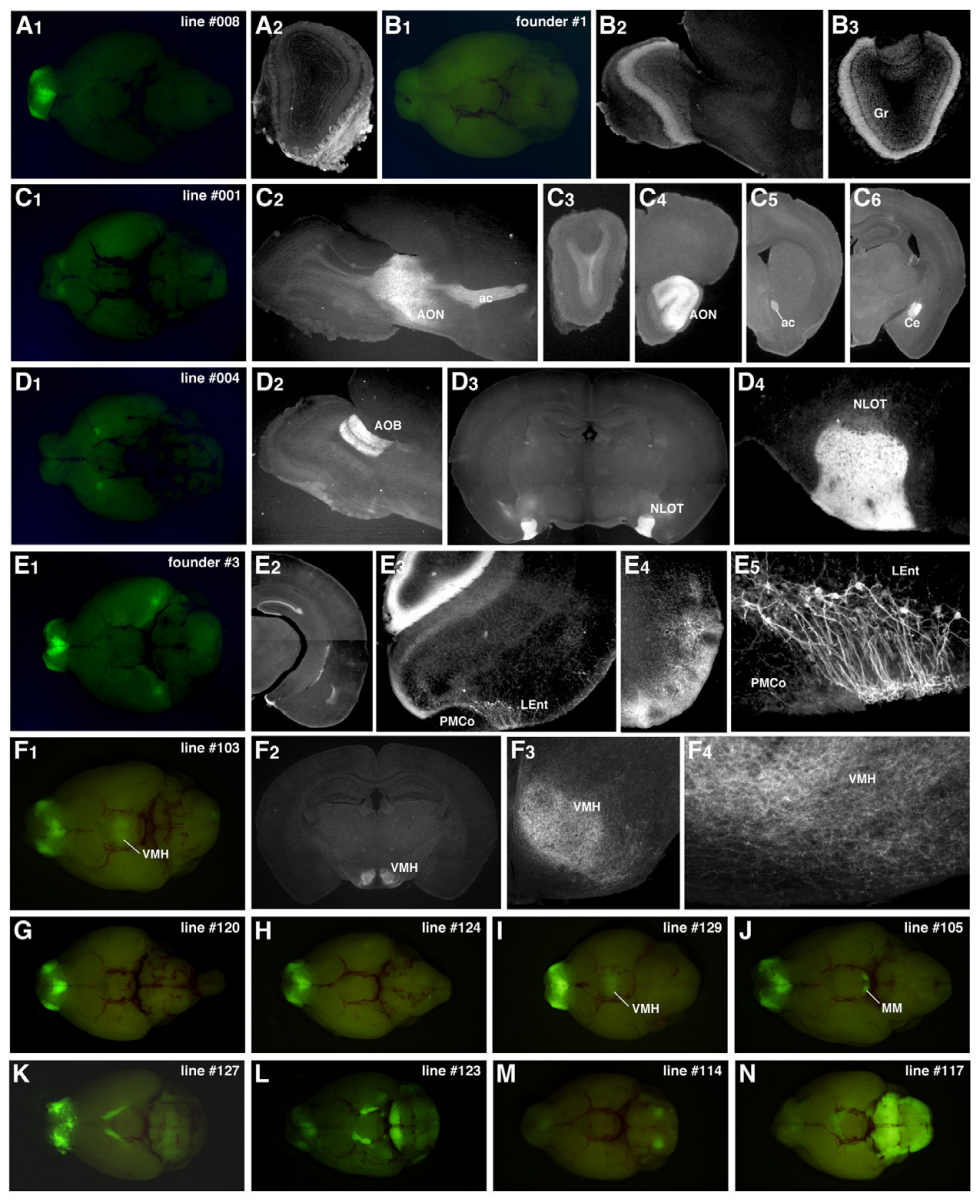
**Variable patterns of transgene expression in Tbx2.6gV transgenic mice**. (A_1_, A_2_) Line #008: a zonal subset of olfactory sensory neurons (OSNs) projecting axons to the ventrolateral region of the olfactory bulb (OB). (B_1_-B_3_) Founder #1: the granule cells (Gr) in the main OB. (C_1_-C_6_) Line #001: the neurons in the anterior olfactory nucleus (AON) projecting axons to the contralateral AON and OB via the anterior commissure (ac) (C_2_-C_5_) and the neurons in the central nucleus of amygdala (Ce) (C_6_). (D_1_-D_4_) Line #004: the granule cells in the accessory OB (AOB) (D_2_) and the neurons in the nucleus of lateral olfactory tract (NLOT) (D_3_, D_4_). (E_1_-E_5_) Founder #3: a small population of neurons in the lateral entorhinal cortex (LEnt) projecting axons to the dentate gyrus of the hippocampus (E_2_-E_5_) and a ventrolateral zonal subset of OSNs. PMCo, posteromedial cortical amygdaloid nucleus. (F_1_-F_4_) Line #103: the neurons in the ventromedial hypothalamic nucleus (VMH) (F_2_-F_4_) and a ventrolateral zonal subset of OSNs. (G-N) Other representative examples of Tbx2.6gV transgenic lines with various patterns of gapVenus expression. MM, medial mammillary nucleus.

Next, we examined Tbx2.6gV transgenic mice in which a 2.6-kb 5'-flanking region was used to direct gapVenus expression. The analysis of Tbx2.6gV mice yielded two interesting findings. First, the transgene expression was hardly detectable in the mitral and tufted cells (2/43: 5%). Among the four founders and 39 lines analysed, only two lines (#127: Figure 4K and #123: Figure 4L, Additional File 2) showed a relatively broad pattern of gapVenus expression in many types of neurons in the brain, including the mitral and tufted cells. Second, the transgene expression was frequently observed in various types of neurons along the olfactory neural pathways (27/43: 63%), although the expression patterns and/or levels differed from line to line. For instance, gapVenus was expressed in the OSNs in the olfactory epithelium in two founders and 21 lines (Figure 4A, E_1_, F_1_, G-M, Additional File 4), among which one founder and six lines also showed the transgene expression in the vomeronasal sensory neurons in the vomeronasal organ. In most of the lines with ectopic transgene expression in OSNs, gapVenus was observed in a restricted subset of OSNs in the ventrolateral zone of the olfactory epithelium, which project axons to the ventrolateral OB (Figure 4A, E_1_, F_1_, G-M, Additional File 4). This pattern of gapVenus expression in the primary olfactory pathway was complementary to that of NAD(P)H:quinone oxidoreductase **(**NQO1)-positive OSNs in the dorsomedial zone (Additional File 4). Other examples of gapVenus expression along the olfactory neural pathways included the granule cells of OB (founder #1: Figure 4B), the neurons in the anterior olfactory nucleus (line #1: Figure 4C), the neurons in the nucleus of lateral olfactory tract and the granule cells in AOB (line #4: Figure 4D), a subset of neurons in the lateral entorhinal cortex (founder #3: Figure 4E) and the neurons in the ventromedial hypothalamic nucleus (line #103: Figure 4F, line #129: Figure 4I). In several lines which showed labelling of certain types of neurons along the olfactory pathways, the transgene was also expressed in cells of non-olfactory-related areas (Additional File 2) such as the mammillary nucleus (line #105: Figure 4J), the pontine nucleus and the optic nerve (line #123: Figure 4L), the facial nucleus (line #114: Figure 4M), the thalamus, the hippocampus, the cerebellum, the area postrema and the spinal cord (data not shown). In only two instances, gapVenus expression was detected restrictedly in non-olfactory-related areas: the cerebellum (founder #4: data not shown) and the brainstem oligodendrocytes and the optic nerve (line #117: Figure 4N). These results suggest that the 2.6-kb 5'-flanking region of *Tbx21 *gene does not contain the mitral and tufted cell enhancer but can drive the transgene expression preferentially in some neurons along the olfactory neural pathways.

In contrast, the shortest fragment with 1.0-kb upstream region of the *Tbx21 *gene showed little enhancer/promoter activity. In all three lines of Tbx1.0gV transgenic mice, gapVenus was not detectable in any type of cells along the olfactory neural pathways including the mitral and tufted cells (Additional File 2).

### MCE: a transcriptional enhancer for transgene expression in mitral and tufted cells

**Figure 5:**
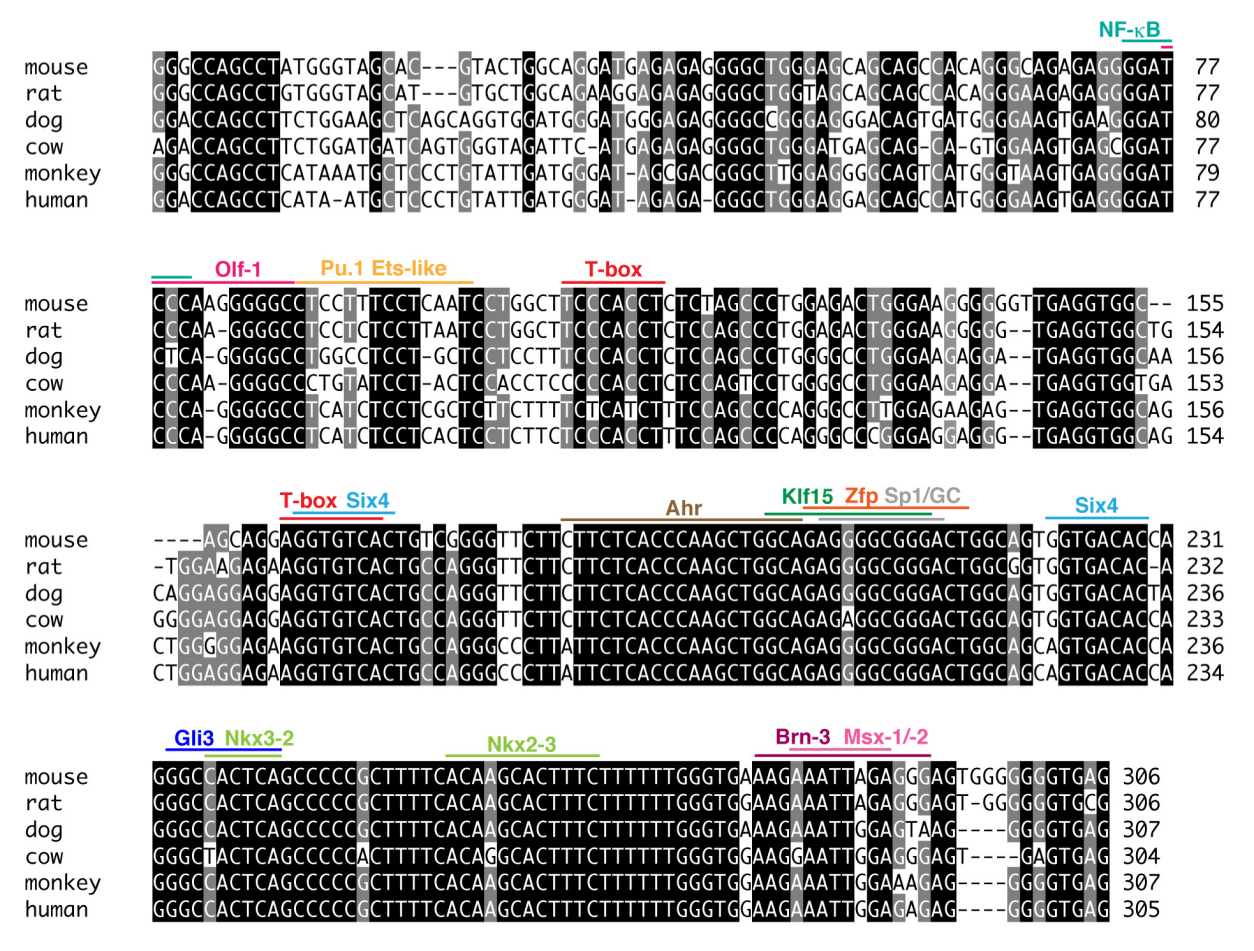
**Nucleotide sequence alignment of mitral and tufted cell-specific enhancer (MCE) from various mammalian species**. Nucleotide sequence alignment of MCE from mouse, rat, dog, cow, monkey and human Tbx21 gene upstream regions. Putative transcription factor-binding sites are shown with upper bars. Two T-box sites (red) are present, which may act as targets of Tbr1, Tbr2 or Tbx21 expressed in the mitral and tufted cells.

**Figure 6:**
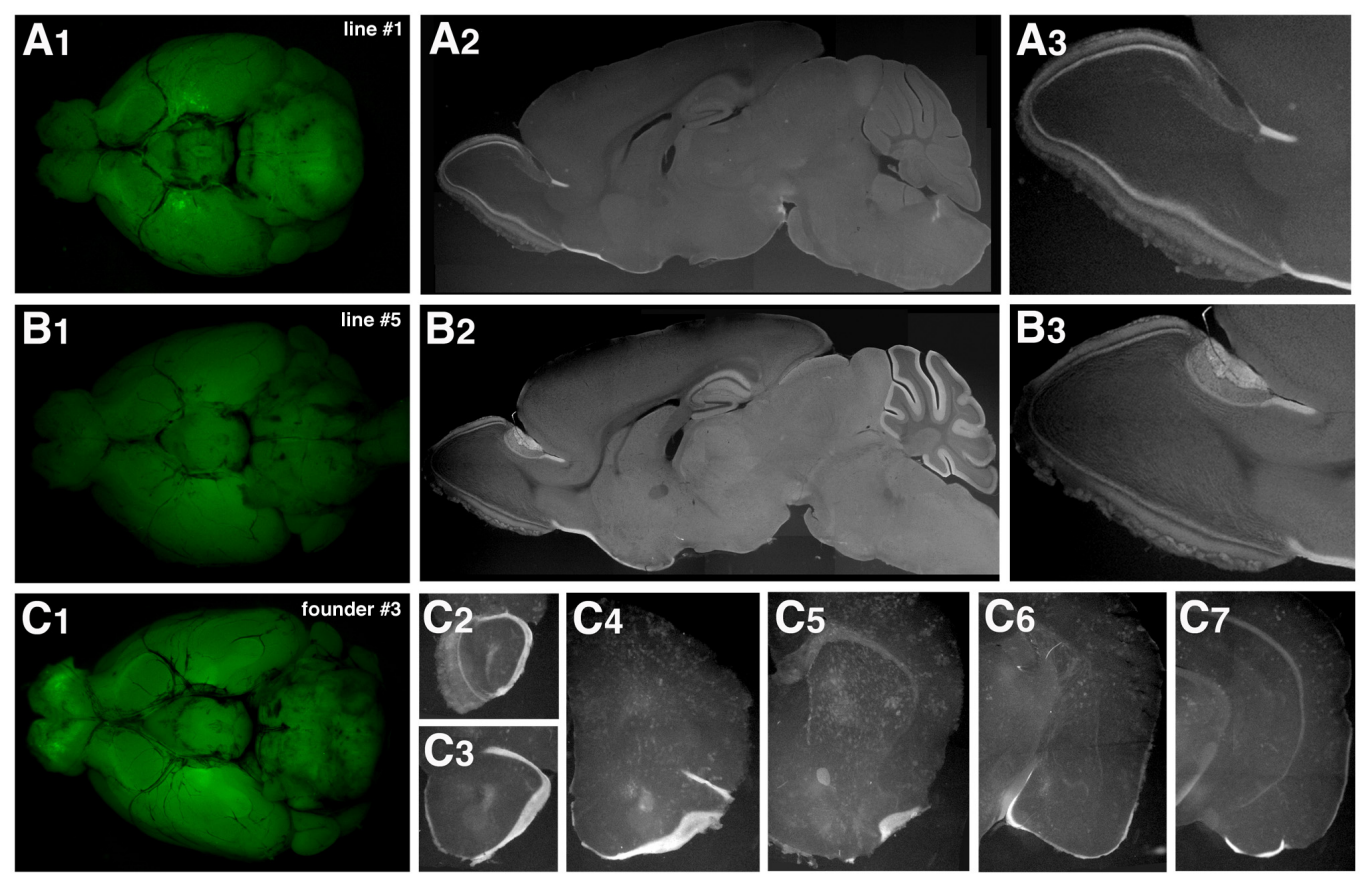
**Mitral and tufted cell-specific enhancer (MCE) directs transgene expression in mitral and tufted cells**. (A_1_-A_3_) MCE-gV transgenic mouse line #1 (A_1_-A_3_), line #5 (B_1_-B_3_) and founder #3 (C_1_-C_7_). (A_1_, B_1_, C_1_) gapVenus fluorescence of whole-mount brains viewed from ventral side. (A_2_, B_2_) Parasagittal brain sections of MCE-gV lines #1 (A_2_) and #5 (B_2_ ) labelled with anti-green fluorescent protein (GFP) antibody. (A_3_, B_3_) Magnified views of the olfactory bulb. (C_2_-C_7_) Coronal sections of MCE-gV founder #3 labelled with anti-GFP antibody. In these three MCE-gV transgenic lines, gapVenus expression is detected in the mitral and tufted cells.

The above results indicate that a crucial *cis*-regulatory element for the transcription in mitral and tufted cells resides between -5.0 and -2.6 kb from the transcription start site of mouse *Tbx21 *gene. By a scrutinized homology search, we identified an extremely conserved 307-bp sequence (putative mitral and tufted cell-specific enhancer; MCE) at ~ 3.0 kb upstream of the transcription start site of the mouse *Tbx21 *gene, which showed more than 80% nucleotide identities with the human, monkey, dog, and rat orthologs (Figure 2B and 5). In order to examine whether this region can act as a transcriptional enhancer to direct gene expression in the mitral and tufted cells, we constructed a transgene consisting of MCE, SV40 minimal promoter, gapVenus and SV40 polyadenylation signal (Figure 2C: MCE-gV) and generated transgenic mice. Among the three founders and seven lines of MCE-gV transgenic mice examined, gapVenus expression was observed in the mitral and tufted cells in the line #1 (Figure 6A), line #5 (Figure 6B), founder #3 (Figure 6C) and line #8 (data not shown; 4/10: 40%, Additional File 2). Although the fluorescence intensity of gapVenus in the mitral and tufted cells was weak on whole-mount brain preparations (Figure 6 A_1_, B_1 _and C_1_) and ectopic transgene expression was frequently observed in other types of cells in these lines, anti-GFP immunohistochemistry of the brain sections clearly demonstrated the transgene expression in the mitral and tufted cells (Figure 6A_2,3_, B_2, 3 _and C_2-7_). Together with the results of Tbx5.0gV, Tbx2.6gV and Tbx1.0gV transgenic mice, these findings indicate that the conserved sequence of 307 bp, designated as MCE, is required for and capable of inducing transgene expression in the mitral and tufted cells.

### Transgenic mouse expressing spH in mitral and tufted cells

**Figure 7:**
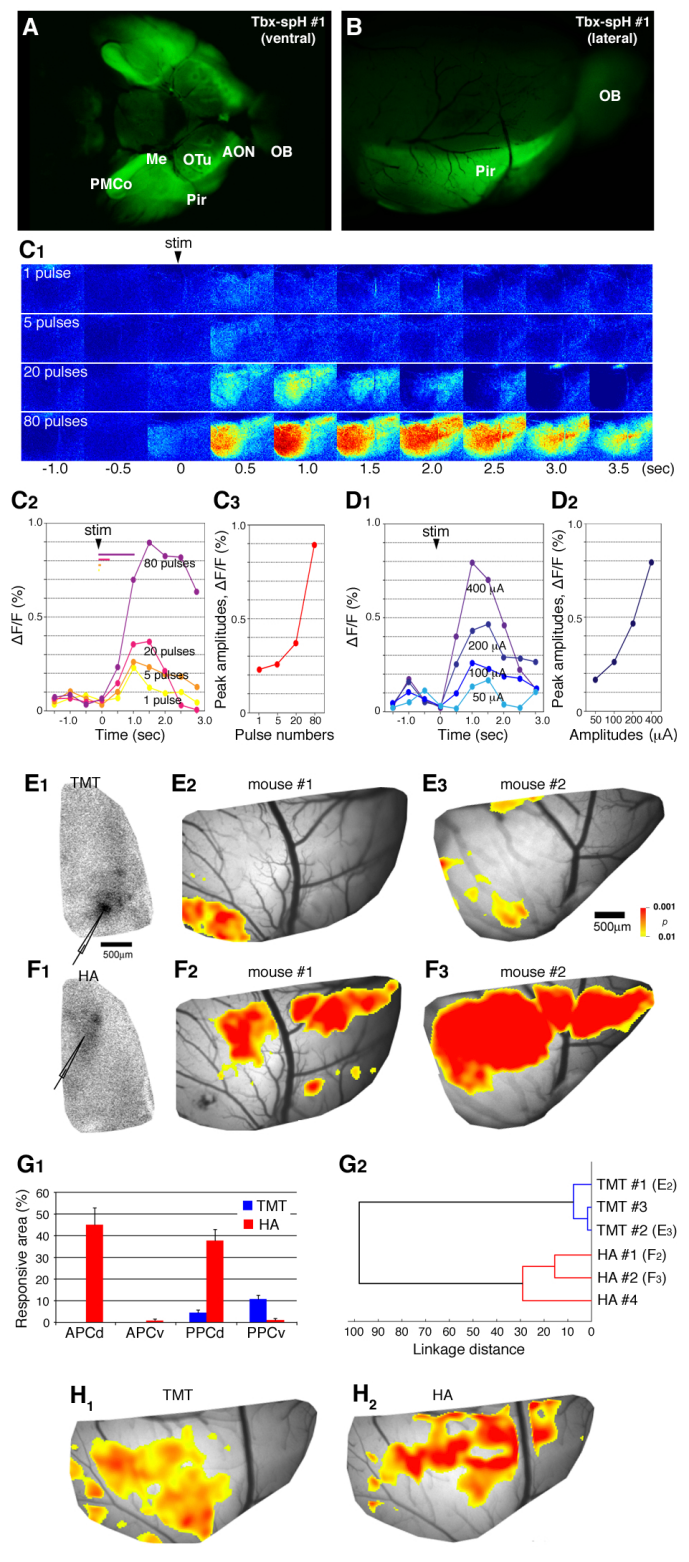
**Transgenic mouse expressing synaptopHluorin (spH) in mitral and tufted cells**. (A, B) Fluorescence of whole-mount brain of Tbx-spH transgenic mouse viewed from ventral (A) and lateral side (B). (C_1_-C_3_) spH signal increase in the piriform cortex is dependent on the pulse numbers of electrical stimulation onto olfactory bulb (OB) glomeruli. ΔF/F fluorescence change images (C_1_), time course (C_2_) and pulse-response relationship (C_3_). (D_1_, D_2_) spH signal increase is dependent on the amplitude of electrical stimulation onto OB glomeruli. Time course (D_1_) and amplitude-response relationship (D_2_). (E_1_-F_3_) spH signal increase upon the electrical stimulation of trimethylthiazoline (TMT; E_1_-E_3_) and heptanoic acid (HA)-responsive (F_1_-F_3_) glomeruli. Odourant-responsive glomeruli were identified by the intrinsic signal imaging of the dorsal surface of OB and stimulated with a microelectrode (E_1_, F_1_). The electrical stimulation of these two glomeruli leads to the spH signal increase in distinct subregions of the piriform cortex (E_2_, E_3_, F_2_, F_3_). (G_1_) Similarity and dissimilarity between fluorescent responses evoked by the OB electrical microstimulation. The imaged region was divided into four subregions (APCd, APCv, PPCd, PPCv: dorsal and ventral parts of the anterior and posterior piriform cortex) [40,43]. Significantly responsive areas (*P *< 0.01, yellow-to-red areas in E_2-3 _and F_2-3_) evoked by electrical stimulation of TMT- and HA-responsive glomeruli were plotted (*n *= 3). (G_2_) Cluster analysis of spH responses. The linkage distance was 4.95 ± 1.70 between any two TMT stimulation images and 22.4 ± 4.53 between any two HA stimulation images. In contrast, the distance between any TMT stimulation image and any HA stimulation image was significantly larger (58.0 ± 1.95; *P*< 0.00001). (H_1_, H_2_) spH signal increase in the piriform cortex upon odourant stimulation. TMT (H_1_) or HA (H_2_) applied into mouse nostril leads to the spH signal increase in overlapping but distinct regions of the piriform cortex.

Finally, we made use of the Tbx21 enhancer/promoter for targeted expression of spH in the mitral and tufted cells. spH is a genetically-encoded fluorescent protein which can be used as an indicator of exocytosis, reflecting the presynaptic neural activities [20-22]. Five transgenic mouse lines (Tbx-spH) were generated that harbour the 5.0-kb Tbx21 enhancer/promoter region fused to spH (Figure 2C). Among them, one line (Tbx-spH #1) showed significant fluorescence of spH in mitral and tufted cell axons and their terminal fields in the olfactory cortex. Although a single Tbx-spH allele on heterozygous background showed only weak fluorescence, the generation of homozygous transgenic mice resulted in a marked increase of spH fluorescence that was easily observed on a whole-mount brain preparation under a fluorescence stereomicroscope (Figure 7A and 7B). Therefore, the homozygous Tbx-spH #1 transgenic mice were used in the subsequent *in vivo *experiments.

The piriform cortex was exposed in anaesthetized transgenic mice and the spH fluorescence was monitored with a CCD camera on a conventional fluorescence microscope connected to an image analysis system. In order to validate that the change in spH fluorescence reflects pre-synaptic activity of mitral and tufted cell axon terminals, electrical stimulation was focally applied to a small spot in the glomerular layer of the OB where the dendrites of mitral and tufted cells receive odour information from the OSNs. The intensity of spH fluorescence in the piriform cortex was dependent on the pulse number (Figure 7 C_1_, C_2 _and C_3_) and amplitude (Figure 7 7D_1 _and D_2_) of electrical stimulation to the OB, indicating the positive correlation between the extent of activated mitral and tufted cells and the spH signal intensity in axon terminals of the mitral and tufted cells.

In order to examine how the glomerular map in OB is represented in the piriform cortex, we first identified a glomerulus responsive to specific odourants by using the optical imaging method of intrinsic signals, then applied electrical stimuli onto the glomerulus and compared the pre-synaptic neural activities of the mitral and tufted cells in the piriform cortex. As odourant stimuli, we used two compounds: 2,4,5-trimethylthiazoline (TMT: a component of fox odour) and heptanoic acid (HA: a component of apple odour). TMT activates a few glomeruli in the posterior region (class II domain) of the dorsal zone (Figure 7E_1_), while HA activates glomeruli in the anterior region (class I domain; Figure 77F_1_). Electrical stimulation of these two distinct glomerular foci resulted in the increased spH fluorescence in overlapping, but different, regions of the piriform cortex: the ventroposterior region by TMT (Figure 7E_2 _and E_3_) and the dorsal region by HA (Figure 7F_2 _and F_3_). A quantitative analysis of spH signals revealed that identical stimulations evoked similar spatial patterns of spH responses, whereas different stimulations evoked distinct pattern of spH responses in the piriform cortex (Figure 7G_1 _and G_2_).

Finally, we measured the change of spH fluorescence in the mitral and tufted cell axon terminals upon the odourant stimulation. Applications of TMT and HA to mouse nostril induced a significant increase in spH signals in the anterior piriform cortex with partly overlapping but mostly distinct patterns (Figure 7H_1 _and H_2_). The regions activated by TMT and HA applications roughly corresponded to those activated by the electrical stimulation of the respective glomeruli responsive to the two odourants. Further experiments of *in vivo *spH imaging in the olfactory cortex will enhance our understanding of neuroanatomical and functional architecture of the secondary olfactory system.

## Discussion

Members of the T-box family transcription factors play important roles in development and functions of various tissues including the brain [23]. In the OB, Tbr1, Tbr2 and Tbx21 are expressed in the excitatory projection neurons, mitral and tufted cells [18,24,25]. In particular, the expression of Tbx21 is highly specific to the mitral and tufted cells, whereas the Tbr1 and Tbr2 are also present in various cell types in other brain regions. The strictly localized expression of Tbx21 gene prompted us to analyse its transcriptional enhancer activity using transgenic mouse approach. Transgene expression in the mitral and tufted cells was efficiently induced with the 5'-flanking 5.0-kb region, but neither with the 2.6-kb nor 1.0-kb regions, indicating that the crucial enhancer activity resides within the 5.0 - 2.6 kb upstream sequence (Figure 2 and 3). *In silico *homology search of Tbx21 genes from various animal species identified a highly conserved ~300-bp sequence located at 3.2-kb upstream from the transcriptional start site of mouse Tbx21 gene. When combined with the SV40 minimal promoter, this ~300-bp sequence could direct the transgene expression in the mitral and tufted cells (Figure 6). Hence, we designated this sequence as the mitral and tufted cell enhancer, MCE.

The nucleotide sequences of MCE in various mammalian species are extremely conserved with higher than 80% identities (Figure 5). The MCE contains several characteristic motifs of transcription factor-binding sites. Intriguingly, two putative T-box-binding sites are present in the mouse MCE, which are similar to the consensus T-box motif (TCACACCT) with only one nucleotide substitution (Figure 5) [23,26], suggesting a possibility that Tbx21 gene expression is regulated by T-box transcription factors expressed in the mitral and tufted cells such as Tbr1, Tbr2 or Tbx21 itself. This notion is corroborated by the fact Tbx21 expression is completely absent in the OB of Tbr1-deficient mice [18]. In addition, the MCE contains putative binding sites for Brn-3, Gli3, Klf15 and Six4. These transcription factors and related molecules belonging to the same families seem to play important roles in various aspects of the olfactory system development [27-31]. Further studies are required in order to identify the trans-binding factors crucial for the mitral and tufted cell-specific transcriptional regulation by MCE.

Previous studies described transgene expression in the mitral and tufted cells by using transcriptional enhancers of Pcdh21 [15], neurotensin [16] and AP-2ε [17]. For neurotensin and AP-2ε, the gene targeting strategy was utilized in order to make knock-in mice expressing tauGFP and Cre recombinase, respectively. For Pcdh21, a long upstream region encompassing ~10 kb was required for the expression of Cre and tetracycline activator in transgenic mice. However, in the cases of Pcdh21 and neurotensin, the transgene expression is not specific to the mitral and tufted cells but is also observed in other types of neurons in the brain [15,16]. For AP-2ε, the transgene expressions in the mitral and tufted cells do not persist into adulthood but begin to diminish soon after birth [17,32]. Thus, the Tbx21 gene enhancer/promoter will become a more useful and convenient tool for genetic manipulations of the mitral and tufted cells because its highly specific and efficient enhancer activity with the shorter length of necessary sequence (~5 kb) guarantees the mitral and tufted cell-directed transgene expression persistently into adulthood.

Unexpectedly, we observed curious patterns of gapVenus expression in many transgenic founders and lines, when the 2.6-kb upstream region of Tbx21 gene was used as the enhancer/promoter. In many lines of Tbx2.6gV transgenic mice, gapVenus expression was detected frequently in various types of neurons along the olfactory neural pathways, such as the OSNs, vomeronasal sensory neurons, granule cells in the OB, neurons in the olfactory cortex including the anterior olfactory nucleus, piriform cortex, nucleus of the lateral olfactory tract, lateral entorhinal cortex and the ventromedial hypothalamic nucleus. We have encountered a similar phenomenon with transgene zebrafish in which a 10-kb upstream region of *Lhx2a *gene was used as an enhancer/promoter. Although the endogenous *Lhx2a *gene is specifically expressed in a small subset of OSNs in zebrafish, fluorescent reporters were detected faithfully not only in the OSNs but also in a subset of mitral cells ectopically [33]. Interestingly, the fluorescent OSNs projected their axons predominantly into a glomerular cluster onto which the fluorescent mitral cells send their dendrites, suggesting that the transgene was coincidently expressed in the pre-synaptic and post-synaptic neurons. Thus, we speculate the existence of a putative transcriptional regulatory system that is active along selective neural circuits. Such a mechanism would, for example, help coordinated expression of homophilic adhesion molecules in both pre- and post-synaptic neurons for the formation of synaptic connections. Further studies are in progress to identify the olfactory circuit-specific transcriptional enhancer elements in both mice and zebrafish and to elucidate their evolutional and physiological significance of regulatory gene expression along selective neural circuits.

We have applied the MCE-containing Tbx21 enhancer for specific expression of spH in the mitral and tufted cells and succeeded in *in vivo *imaging of presynaptic activities in the piriform cortex of transgenic mice. Upon either the electrical stimulation of OB glomeruli or the odourant application into nostril, we observed the significant increase in spH signals in the piriform cortex, reflecting the active exocytosis events at the axon termini of the mitral and tufted cells. Further comprehensive analysis with a systematic battery of various odour molecules will help us to decipher how the odour information represented on the glomerular array of OB is transferred to the third-order neurons in the olfactory cortex. In addition to spH, any genetically-encoded molecular tools of interest will be expressed in the mitral and tufted cells by the use of Tbx21 enhancer.

## Conclusions

We have identified the mitral and tufted cell-specific transcriptional enhancer upstream of the mouse Tbx21 gene. A short sequence of ~300 bp was necessary and sufficient for the transgene expression in these projection neurons of the olfactory bulb. spH expression in the mitral and tufted cells enabled us to monitor the presynaptic neural activities in the piriform cortex. Thus, this study paves a new avenue of the olfactory research into higher brain centres.

## Methods

### Plasmid construction

The membrane-targeted fluorescent reporter, gapVenus, consists of a variant of yellow fluorescent protein with fast and efficient maturation (Venus) and an N-terminal palmitoylation signal of growth-associated protein-43 (GAP-43) [34,35]. gapVenus cDNA was inserted into *EcoRI *site of pBstN vector (containing human β-globin gene intron and Simian virus 40 (SV40) polyadenylation signal) to construct pBstN-gapVenus. The 5.0-kb 5'-flanking region of mouse Tbx21 gene was amplified from mouse tail DNA as a template with a pair of primers (F002-*SacII*: 5'-TTCCCCGCGGATAAGTGGCCTGACGTACAGGCGAG-3'; R002-*XbaI*: 5'-ATTCTCTAGACCGAGGCGGGCTGGGCGCCTTCCAG-3') using Expand High Fidelity polymerase chain reaction (PCR) System (Roche Applied Science, IN, USA) and inserted into the *SacII/XbaI *site of pBstN-gapVenus to generate pTbx5.0gV. Tbx5.0gV, Tbx2.6gV and Tbx1.0gV transgenes with different lengths of the Tbx21 promoter/enhancer region were excised form pTbx5.0gV plasmid by digestion with *SacII/KpnI*, *EcoRV/KpnI *and *AgeI/KpnI*, respectively. In order to construct pMCE-gV, a 307-bp fragment (-3462 ~ -3045) located upstream of the transcription initiation site of mouse Tbx21 gene was PCR-amplified and inserted into pBstN-gapVenus plasmid. In order to construct pTbx-spH, a similar procedure was used as described above for pTbx5.0gV, except that cDNA encoding spH [20] was used instead of gapVenus.

### Animals

The generation of transgenic mice was performed as previously described [36]. Briefly, gel-purified transgenes were microinjected into the pronucleus of fertilized eggs that were obtained from crossing (C56BL/6J × DBA/2J) F_1 _mice. The manipulated eggs were cultured to the two-cell stage and transferred into oviducts of pseudo-pregnant foster females (ICR strain). Integration of the transgenes was screened by PCR of tail DNA. The *Tbx21 (T-bet) *mutant mice [37] were purchased from The Jackson Laboratory (Maine, USA). All animal experiments were approved by the Animal Care and Use Committee of RIKEN and conformed to National Institutes of Health guidelines.

### Immunohistochemistry

Immunohistochemistry of brain sections was carried out as previously described [38]. The following primary antibodies were used: guinea pig anti-Tbx21 (1:10000) [19]; rat anti-GFP (1:1000, Nacalai Tesque, Kyoto, Japan); rabbit anti-Arx (1:1000 provided by Dr K Kitamura) [39]; rabbit anti-Tbr1 (1:1000, Abcam, Cambridge, UK), rat phycoerythrin-conjugated anti-Tbr2 (1:200, eBioscience, CA, USA), rabbit anti-Pcdh21 (1:1000) [14]; mouse anti-PGP9.5 (1:100, Abcam); goat anti-NQO1 (1:100, Abcam). Secondary antibodies labelled with Cy3, Cy5 or Alexa488 were purchased from Jackson ImmunoResearch (PA, USA) and Molecular Probes (OR, USA). Fluorescent images were obtained with a fluorescent microscope (Axioplan, Carl Zeiss, Oberkochen, Germany) equipped with a CCD camera and an image analysis system (DP-70, Olympus, Tokyo, Japan) or a confocal laser scanning microscope (Fluoview FV1000, Olympus).

### *In vivo *spH imaging

Imaging was performed in homozygous Tbx-spH mice aged 6-7 weeks in accordance with the guidelines of the Physiological Society of Japan and the animal experiment committee of the University of Tokyo. Animals were anaesthetized by intraperitoneal injection of medetomidine (0.5 mg/kg) and urethane (0.6 g/kg). Additional doses of urethane were given if necessary. Animals were placed in a custom-built stereotaxic apparatus (Narishige, Tokyo, Japan). Body temperature was maintained at 37.5°C using a homeothermic heatpad system (MK-900, Muromachi, Tokyo, Japan). Respiratory rhythms were detected using a piezo transducer (MLT 1010, ADInstruments Japan Inc, Nagoya, Japan). The anterior and posterior piriform cortex was exposed by removing a 5 × 4 mm area of skull as previously described [40]. Agarose gel was mounted on the brain and covered with a glass coverslip to form a temporary chamber for the optical imaging. The piriform cortex was imaged using an Olympus BX51 microscope and epifluorescence condenser, with 4 × (0.28 NA) Olympus objective. Illumination was provided by 75W Xenon arc lamp (Olympus). A filter set of 490-500HP (exciter), 505 (dichroic) and 515-560HQ (emitter) was used. Optical signals were recorded using a cooled CCD camera (ORCA-ER, Hamamatsu Photonics, Hamamatsu, Japan) at 336 × 256 pixel resolution and a frame rate of 5 Hz. Data acquisition was performed with a digital frame grabber board (NI PCI-1424, National Instruments, TX, USA) controlled by custom software written in LabVIEW (National Instruments). Image acquisition was post-triggered by an initiation of respiration using a custom-built circuit so that the acquisition of a first post-stimulus frame was always time-locked with respiration initiation.

Electrical microstimulation (100 μA, 60 Hz, 5 pulses of 0.2 ms, except for stimulus-response experiments) was performed using an iridium electrode (MicroProbes, MD, USA), a stimulator (Master-8, AMPI, Jerusalem, Israel) and a stimulus isolator (SS501J, Nihon Kohden, Tokyo, Japan). For odour stimulations, 10% dilutions (in mineral oil) of TMT (Contech Inc, Delta, Canada) and HA (Tokyo Kasei, Tokyo, Japan) were used. Odourants were delivered using a computer-controlled custom-built olfactometer. Initiations of microstimulation and odour stimulation were time-locked with the respiration initiation that also triggered the acquisition of the first post-stimulus fame.

Images were analysed using IDL (Research Systems, CO, USA) software. Raw traces were corrected for photobleaching by subtracting no-stimulus trials before further analysis [21]. A differential image was obtained by dividing the temporal average of signals acquired during electrical or odour stimulations (from 0.5 to 1.5 s or from 0.5 to 2.0 s after stimulus onset, respectively) by 2-s temporal average acquired before stimulation. A Gaussian spatial filter was used to eliminate nonspecific global fluctuation and high-frequency shot noise of the differential image (cutoff frequencies, σ = 50.0/mm for the high cut-off and σ = 0.1/mm for the low cut-off). In order to achieve a better signal-to-noise ratio, these filtered images were averaged for 10 trials. The pixel intensities of the images with stimulus were compared by pixel-by-pixel *t*-test (*P *< 0.01, two-tailed *t*-test, *n *= 10 trials) with those with blank (pure air) stimulus. Significant pixels were overlaid on the image of the blood vessels. Final images were imported into Adobe Photoshop 6.0 for cropping and display. In stimulus-response experiments, the increase of fluorescence (ΔF/F) was calculated for all exposed regions in the piriform cortex with 0.5 s time bins. spH responses in different parts of the piriform cortex upon glomerular microstimulation were quantified from three mice and subjected to a cluster analysis.

### *In silico *nucleotide sequence analysis

The nucleotide sequence of mouse *Tbx21 *gene was compared with those of human, rhesus, dog and rat orthologs by using the VISTA program (http://genome.lbl.gov/vista/index.shtml, http://pipeline.lbl.gov/cgi-bin/gateway2) [41]. We compared the following sequences. Mouse chromosome 11: 96,956,048 - 96,983,806; human chromosome 17: 43,176,917 - 43,186,235; rhesus chromosome 16: 31,955,925 - 31,961,651; dog chromosome 9: 27,412,274 - 27,418,981; and rat chromosome 10: 85,814,742 - 85,819,229. We used the following default settings of VISTA parameters. Calculation window: 100 base pairs; minimal conserved width: 100 base pairs; and conservation identity: 80%.

Potential transcription factor-binding sites within mouse MCE were predicted with the MatInspector analysis program free trial version (Genomatix, Germany: http://www.genomatix.de/en/index.html) [42]. Selected groups for solution parameters of MatInspector used were as follows: general core promoter elements: 0.75/optimized; vertebrates: 0.75/optimized).

## Abbreviations

AOB: accessory OB; cDNA: complementary DNA; GAP-43: growth-associated protein-43; GFP: green fluorescent protein; HA: heptanoic acid; MCE: mitral and tufted cell-specific enhancer; MOB: main OB; mRNA: messenger RNA; NQO1: NAD(P)H:quinone oxidoreductase; OB: olfactory bulb; OR: odourant receptors; OSN: olfactory sensory neuron; Pcdh21: protocadherin 21; PCR: polymerase chain reaction; spH: synaptopHluorin; SV40: Simian virus 40; TMT: 2,3,5-trimethylthiazoline.

## Competing interests

The authors declare that they have no competing interests.

## Authors' contributions

SM performed the molecular biological and immunohistochemical experiments and drafted the manuscript. KMI and KM carried out the physiological experiments and helped to draft the manuscript. YY conceived the study, carried out molecular biological and immunohistochemical experiments and drafted the manuscript. All authors read and approved the final manuscript.

## Supplementary Material

Additional file 1 **Quantification of numbers of Tbx21-positive mitral and tufted cells in comparison with Tbr1 and protocadhein 21 (Pcdh21)**. Five coronal sections at different levels along the anteroposterior axis (600 μm interval) were prepared from five olfactory bulbs (OBs) and double-labelled with anti-Tbx21 and anti-Tbr1 antibodies or with anti-Tbx21 and anti-Pcdh21 antibodies. For mitral cells, the numbers of Tbx21- and/or Tbr1-positive cells in the mitral cell layer were counted. For tufted cells, the numbers of Tbx21- and/or Pcdh21-positive cells in the external plexiform layer were counted. Tbx21 is expressed in a vast majority, if not all, of mitral cells (94.6 ± 0.6% of the Tbr1-positive mitral cells) and in a large population of tufted cells (72.4 ± 3.4% of the Pcdh21-positive tufted cells). Click here for file

Additional file 2 **Summary of gapVenus expression in Tbx21 transgenic mice**. Transgenic founders and lines with neuronal gapVenus expression are summarized. Highly efficient rate of gapVenus expression in the mitral and tufted cells is obvious in Tbx5.0gV founders and lines. On the other hand, variable patterns of gapVenus expression are observed among Tbx2.6gV founders and lines. Especially, most of the Tbx2.6gV founders and lines show gapVenus expression in some neurons along the olfactory neural pathways. gapVenus expression in mitral and tufted cells is detected in four of the five MCE-gV founder and lines. Red squares indicate the presence of gapVenus-expressing neurons in olfactory-related regions (dark red) and other brain regions (light red); light blue squares indicate gapVenus-expressing non-neuronal cells. M/T, miral and tufted cells; Gr, granule cells; OC, olfactory cortex; MOE, main olfactory epithelium; VNO, vomeronasal organ; MOB, main olfactory bulb; AOB, accessory olfactory bulb; AON, anterior olfactory nucleus; OTu, olfactory tubercle; Pir, piriform cortex; NLOT, nucleus of lateral olfactory tract; Amyg, amygdala; LEnt, lateral entorhinal cortex. Click here for file

Additional file 3 **Ontogenic expression of endogenous Tbx21 and transgenic gapVenus proteins in the olfactory bulb (OB) of Tbx5.0gV mice [line #3(2)]**. Double immunofluorescence labelling of OB coronal sections from Tbx5.0gV transgenic mice [line #3(2)] at different developmental stages (A, E, I) E14.5; (B, F, J) E16.5; (C, G, K) P0; (D, H, L) P7 with anti-Tbx21 (A-D (white), I-L (magenta)) and anti-green fluorescent protein (E-H (white), I-L (green)) antibodies. Both the endogenous Tbx21 and transgenic gapVenus proteins first appear in the mitral cells at E14.5 and gradually increase thereafter until P7. As the tufted cells develop much later than the mitral cells, the presence of both proteins in the tufted cells becomes obvious after birth. An apparent dissociation of two immunoreactive signals in the merged images (J-L) results from the different intracellular localization of the two proteins (Tbx21: cellular nuclei, gapVenus: plasma membranes mainly of axons) in the mitral and tufted cells. Click here for file

Additional file 4 **Zonal expression of gapVenus in the primary olfactory pathway of Tbx2.6gV transgenic mouse (line #120)**. Double immunofluorescence labelling of coronal sections of the olfactory epithelium (A-F) and olfactory bulb (G-L) from Tbx2.6gV transgenic mice (line #120) with anti-green fluorescent protein (A, D, G, J (white), C, F, I, L (green)) and anti-NAD(P)H:quinone oxidoreductase (NQO1) (B, E, H, K (white), C, F, I, L (magenta)) antibodies. The regions indicated by rectangles in (A-C) and (G-I) are magnified in (D-F) and (J-L), respectively. The expression of gapVenus is restricted to the ventral zone of the olfactory epithelium, complementary to that of the dorsal zone-specific NQO1. A similar pattern of gapVenus expression is observed in other Tbx2.6gV transgenic lines (#006, 008, 103, 105, 124, 127 and 129; data not shown). Click here for file
