# Supplementary material for: Genetic visualization of the secondary olfactory pathway in Tbx21 transgenic mice
Source: Neural Syst Circuits. 2011 Feb 1;1:5. doi: 10.1186/2042-1001-1-5 (PMC3257540; doi:10.1186/2042-1001-1-5)
Supplement: Additional file 1 — Quantification of numbers of Tbx21-positive mitral and tufted cells in comparison with Tbr1 and protocadhein 21 (Pcdh21). Five coronal sections at different levels along the anteroposterior axis (600 μm interval) were prepared from five olfactory bulbs (OBs) and double-labelled with anti-Tbx21 and anti-Tbr1 antibodies or with anti-Tbx21 and anti-Pcdh21 antibodies. For mitral cells, the numbers of Tbx21- and/or Tbr1-positive cells in the mitral cell layer were counted. For tufted cells, the numbers of Tbx21- and/or Pcdh21-positive cells in the external plexiform layer were counted. Tbx21 is expressed in a vast majority, if not all, of mitral cells (94.6 ± 0.6% of the Tbr1-positive mitral cells) and in a large population of tufted cells (72.4 ± 3.4% of the Pcdh21-positive tufted cells). [file 2042-1001-1-5-S1.PDF]

anterior ↕ posterior

Mitral cells

Section	Tbx21	Tbr1	Mean ± SEM
#1	+	+	310 ± 17 (94.2 ± 1.2%)
	+	−	9 ± 2 (2.7 ± 0.5%)
	−	+	10 ± 2 (3.1 ± 0.7%)
#2	+	+	401 ± 27 (94.2 ± 0.8%)
	+	−	13 ± 3 (2.9 ± 0.4%)
	−	+	12 ± 2 (2.9 ± 0.5%)
#3	+	+	485 ± 27 (95.8 ± 0.5%)
	+	−	10 ± 2 (1.9 ± 0.3%)
	−	+	11 ± 2 (2.2 ± 0.3%)
#4	+	+	416 ± 43 (93.8 ± 1.2%)
	+	−	10 ± 1 (2.3 ± 0.4%)
	−	+	16 ± 3 (3.9 ± 0.9%)
#5	+	+	196 ± 13 (93.5 ± 0.8%)
	+	−	6 ± 2 (3.2 ± 0.9%)
	−	+	7 ± 1 (3.3 ± 0.5%)
Sum	+	+	1808 ± 60 (94.6 ± 0.6%)
	+	−	48 ± 6 (2.5 ± 0.3%)
	−	+	57 ± 9 (2.9 ± 0.4%)

Tufted cells

Section	Tbx21	Pcdh21	Mean ± SEM
#1	+	+	388 ± 60 (75.1 ± 7.4%)
	+	−	2 ± 1 (0.3 ± 0.2%)
	−	+	118 ± 25 (24.7 ± 7.5%)
#2	+	+	589 ± 53 (70.6 ± 4.4%)
	+	−	4 ± 2 (0.5 ± 0.3%)
	−	+	237 ± 31 (28.9 ± 4.7%)
#3	+	+	636 ± 46 (72.1 ± 3.6%)
	+	−	7 ± 3 (0.8 ± 0.3%)
	−	+	237 ± 30 (27.2 ± 3.9%)
#4	+	+	478 ± 62 (72.9 ± 2.9%)
	+	−	11 ± 5 (1.6 ± 0.7%)
	−	+	160 ± 8 (25.4 ± 3.1%)
#5	+	+	241 ± 35 (70.6 ± 2.9%)
	+	−	4 ± 2 (1.1 ± 0.6%)
	−	+	92 ± 5 (28.3 ± 3.4%)
Sum	+	+	2332 ± 204 (72.4 ± 3.4%)
	+	−	28 ± 12 (0.8 ± 0.3%)
	−	+	844 ± 82 (26.8 ± 3.7%)
