# Supplementary figures and images for: Genetic visualization of the secondary olfactory pathway in Tbx21 transgenic mice
Source: Neural Syst Circuits. 2011 Feb 1;1:5. doi: 10.1186/2042-1001-1-5 (PMC3257540; doi:10.1186/2042-1001-1-5)

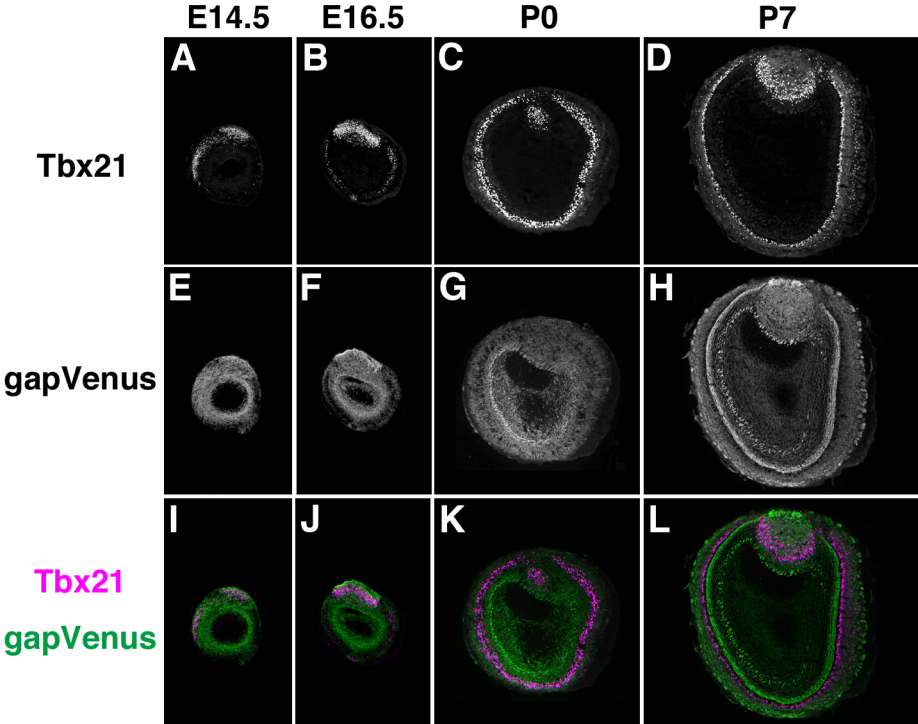

Supplement: Additional file 3 — Ontogenic expression of endogenous Tbx21 and transgenic gapVenus proteins in the olfactory bulb (OB) of Tbx5.0gV mice [line #3(2)]. Double immunofluorescence labelling of OB coronal sections from Tbx5.0gV transgenic mice [line #3(2)] at different developmental stages (A, E, I) E14.5; (B, F, J) E16.5; (C, G, K) P0; (D, H, L) P7 with anti-Tbx21 (A-D (white), I-L (magenta)) and anti-green fluorescent protein (E-H (white), I-L (green)) antibodies. Both the endogenous Tbx21 and transgenic gapVenus proteins first appear in the mitral cells at E14.5 and gradually increase thereafter until P7. As the tufted cells develop much later than the mitral cells, the presence of both proteins in the tufted cells becomes obvious after birth. An apparent dissociation of two immunoreactive signals in the merged images (J-L) results from the different intracellular localization of the two proteins (Tbx21: cellular nuclei, gapVenus: plasma membranes mainly of axons) in the mitral and tufted cells. [file 2042-1001-1-5-S3.PDF]

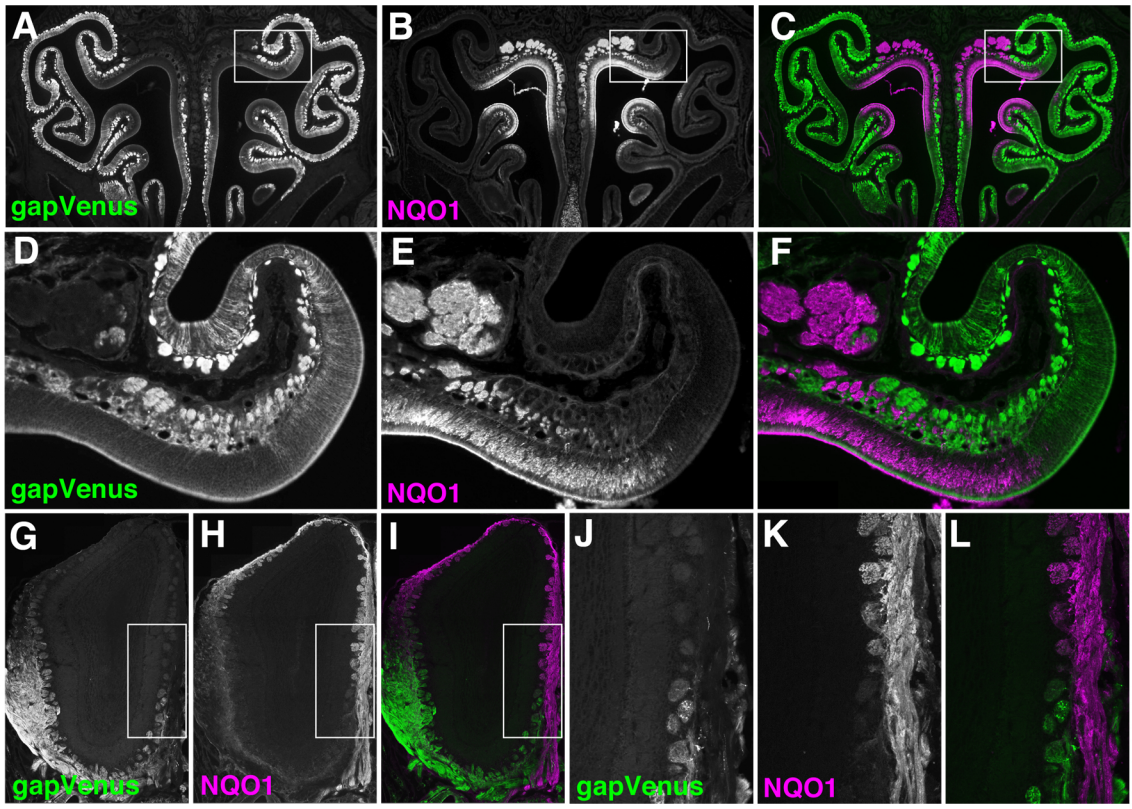

Supplement: Additional file 4 — Zonal expression of gapVenus in the primary olfactory pathway of Tbx2.6gV transgenic mouse (line #120). Double immunofluorescence labelling of coronal sections of the olfactory epithelium (A-F) and olfactory bulb (G-L) from Tbx2.6gV transgenic mice (line #120) with anti-green fluorescent protein (A, D, G, J (white), C, F, I, L (green)) and anti-NAD(P)H:quinone oxidoreductase (NQO1) (B, E, H, K (white), C, F, I, L (magenta)) antibodies. The regions indicated by rectangles in (A-C) and (G-I) are magnified in (D-F) and (J-L), respectively. The expression of gapVenus is restricted to the ventral zone of the olfactory epithelium, complementary to that of the dorsal zone-specific NQO1. A similar pattern of gapVenus expression is observed in other Tbx2.6gV transgenic lines (#006, 008, 103, 105, 124, 127 and 129; data not shown). [file 2042-1001-1-5-S4.PDF]
